# Ultrasonographic features of the platypus abdomen via a limited left pre‐epipubic acoustic window from a series of 54 individuals

**DOI:** 10.1111/avj.70122

**Published:** 2026-07-24

**Authors:** JW Macgregor, RA Lonsdale, C Holyoake, JH Connolly, ID Robertson, R Donaldson, K Warren

**Affiliations:** ^1^ School of Veterinary Medicine, College of Environmental and Life Sciences, Murdoch University Murdoch Western Australia Australia; ^2^ Diagnostic Veterinary Imaging (DVI) Bentley DC Perth Western Australia Australia; ^3^ School of Agricultural, Environmental and Veterinary Sciences, Charles Sturt University Wagga Wagga New South Wales Australia; ^4^ Gulbali Institute, Agriculture, Water and Environment, Charles Sturt University Wagga Wagga New South Wales Australia

**Keywords:** abdomen, Platypus, ultrasonography, wildlife

## Abstract

**Introduction:**

There are notable differences between the platypus and common species of domestic mammal in the location and structure of some abdominal organs. The aim of this study was to detail the size, position and relative locations of organs in the left side of the platypus abdomen, to assist evidence‐based decision‐making in a clinical setting.

**Materials and methods:**

Abdominal ultrasonography was performed on 54 wild, adult platypuses (31 males, 23 females) in northwest Tasmania. Examinations were performed under isoflurane anaesthesia, with platypuses in dorsal recumbency, through a 4 × 2 cm acoustic window between the caudal ribs and the epipubic bone. Abdominal organ position and morphometrics were recorded.

**Results:**

The right ventral process of the spleen was located directly under the acoustic window in 53 platypuses. The left kidney was always located dorsal to the right ventral process of the spleen and, at least in part, cranial to it in 46 platypuses. The left dorsal process of the spleen was located dorsally. The left testis or left ovary was located caudal or caudoventral to the left kidney and caudal or caudodorsal to the right ventral process of the spleen.

**Discussion:**

The described acoustic window on the left ventral abdomen provides good ultrasound access to a number of organs in the left side of the abdomen. The position of the right ventral process of the spleen in most platypuses, directly under this clipped area, makes this structure a good landmark and starting point for ultrasound examination of the other organs imaged.

**Abbreviations**
BV: blood vessel
K: left kidney
LDS: left dorsal process of the spleen
O: left ovary
RVS: right ventral process of the spleen
S: spleen
T: testis
UB: urinary bladder

## Introduction

Ultrasonography was first utilised in veterinary medicine for body condition scoring and reproduction management in domestic animals in the 1950s, followed by the diagnostic imaging of disease; but was slow to be introduced in nondomestic and exotic species.^1^ More recently ultrasonography has been used to characterise abdominal organs in koalas,^2^, ^3^ kangaroos^4^ and echidnas,^5^ however up until now reporting on the use of ultrasonography in the platypus has been limited to a study that imaged tail fat as a measure of body condition.^6^

While the abdominal organs of the platypus, and their positions, are similar to those in common domestic species of mammals, there are some notable differences.^7^, ^8^. The platypus spleen is bi‐lobed, having a left dorsal process and a right ventral process.^9^, ^10^ The female has a left and right ovary; however only the left one is functional and it is only seasonally active.^11^, ^12^ Females have a left and right uterus, independently opening into the urogenital sinus and then the cloaca.^7^, ^13^, ^14^ The male has intra‐abdominal testes located caudal to the kidneys, which can be relatively large considering the size of the animal.^7^, ^15^ In addition to these unique abdominal organ characteristics, the platypus abdomen is relatively well enclosed by skeletal structures. The rib cage extends caudally further than in many mammals^7^, ^16^ and, like marsupials but unlike eutherian mammals, left and right epipubic bones project cranially from the pelvis along the ventral abdominal wall.^17^ Ultrasonography has been used to examine platypus abdominal organs (Russell Jones, personal communication), however no detailed descriptions of ultrasonographic findings have been published for this species. The aim of this prospective study was to use observations made during an associated investigation into reproductive seasonality to detail the size, position and relative positions of selected organs in the left side of the platypus abdomen, as a reference for examinations performed in a clinical setting.

## Materials and methods

Abdominal ultrasonography was performed on 54 wild, adult platypuses (31 males, 23 females), each examined once, captured in the Inglis River catchment (41.06° S, 145.64° E) in northwest Tasmania, Australia between 16/09/2012 and 26/02/2013. The imaging was undertaken as part of a study on seasonal changes in the platypus reproductive tract and a broader study of platypus population health.^18^ All imaging was performed by J.W.M., a veterinarian with a detailed knowledge of platypus anatomy from clinical and research experience as well as review of available literature, under the direct training and external supervision of R.A.L. a veterinary imaging specialist. These examinations were performed under isoflurane anaesthesia as described in Macgregor et al.,^19^ with platypuses in dorsal recumbency. A 4 × 2 cm area of fur was clipped at ~30° from the sagittal plane on the left side of the ventral aspect of the abdomen, between the ribs and epipubic bone (Figure 1). The aims of the associated reproductive tract study dictated that the small, clipped area was on the left side of the abdomen, because in the female platypus only the left ovary is active. To minimise the effects of clipping on thermoregulation after release, only a small area (4 cm × 2 cm) of fur was clipped, and this was only performed for captured platypus between the spring and summer months of September and February. MES Conductive gel (Medical Equipment Services, Melbourne, Victoria) was applied to the skin in the clipped area, to ensure good contact of the transducer with the skin (Figure 1). An Aloka Prosound 2® ultrasound unit (Hitachi Medical Corporation, Tokyo, Japan) with a 4 cm linear transducer was used, at a frequency of 10Mhz. Examinations were started with a 90° angle of incidence to the abdomen, with fanning of the probe as necessary to identify organs and capture images. Images described as being in the sagittal oblique plane were taken in the orientation of the clipped area. Images described as being in the transverse oblique plane were taken across the clipped area. Testes and kidneys were imaged in their longitudinal axis and in an axis perpendicular to longitudinal (described below as the transverse axis).

**Figure 1:**
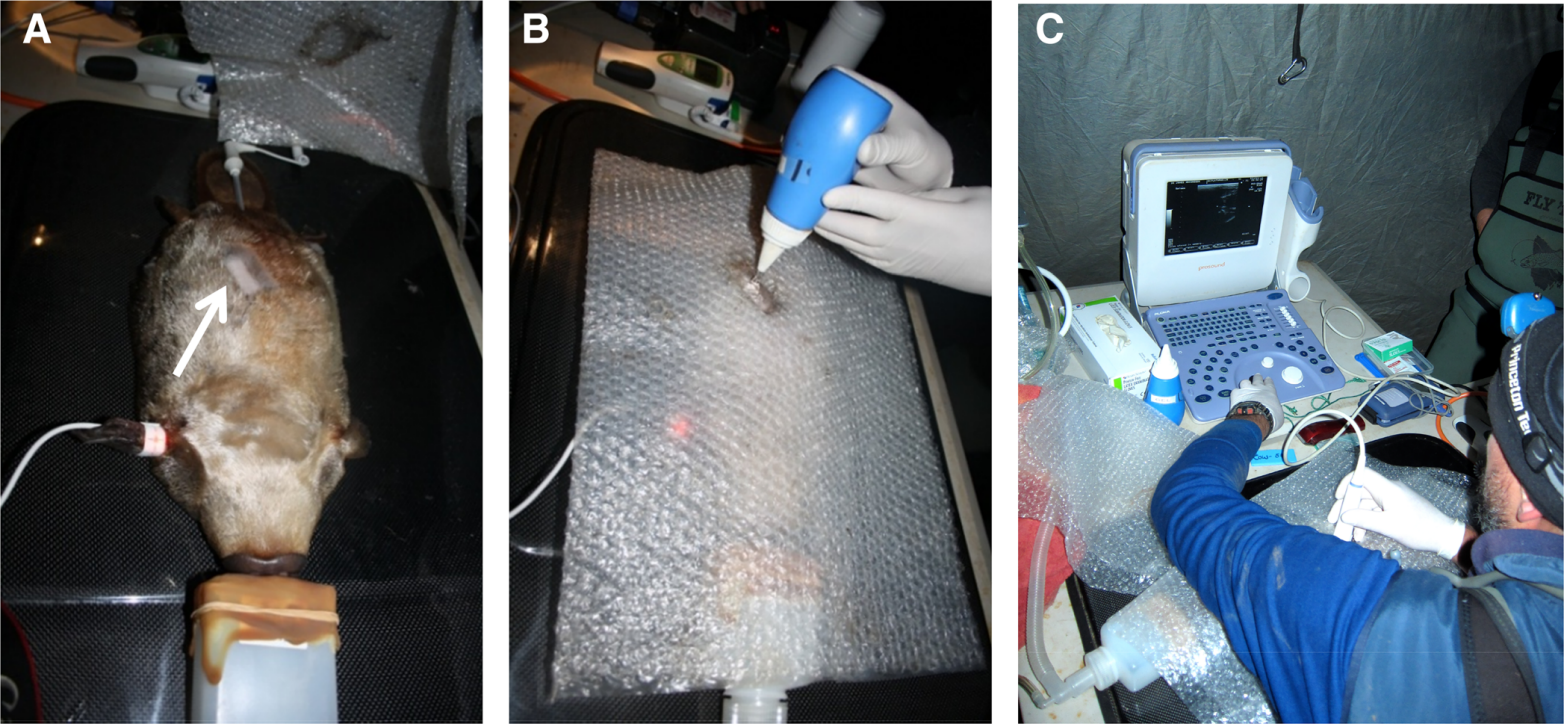
Ultrasonography of the platypus through a pre‐epipubic acoustic window: (A) location of the window of clipped fur (arrow), (B) application of ultrasound gel through hole in bubble wrap blanket, placed over the platypus to minimise heat loss, and (C) ultrasound probe held along the clipped window, at a 90° angle of incidence. Photos: Geoff Dutton.

Measurements of abdominal structures from images were taken using the caliper function on the ultrasound unit. Alternatively, after the ultrasound procedure, the Foxit Phantom pdf distance measure tool (Foxit Software Incorporated, Fremont, CA, USA) was used to measure the distances on the image, after the scale from a known measurement on that image was set.

Data was collated separately for males and females. In individuals where thickness was measured in two perpendicular planes (while measuring length in one view and width in the perpendicular view), an average value was used for statistical analysis. The distributions of measurements with results from greater than two individuals were tested for normality using a Shapiro–Wilk test. Maximum and minimum results for each measurement were determined. Distributions that didn't show departure from normality were expressed by their mean and standard deviation. Distributions that showed a significant departure from normality were expressed by their median and interquartile range. For measurements with results from greater than two of each sex, with both sets of results not deviating from a normal distribution, a *t*‐test was used to investigate whether there was a difference between males and females. For measurements with results from greater than two of each sex, where at least one set of results significantly deviated from a normal distribution, a Mann Whitney *U* test was used to investigate whether the male and female data came from different distributions.

## Results

The right ventral process of the spleen was imaged in the sagittal oblique plane, as a homogeneous, half tear‐drop shaped structure, located adjacent to the ventral body wall in 53 platypuses (Figure 2A). This process of the spleen was usually located directly under the clipped area of skin. A small oval or round anechoic structure, adjacent to the craniodorsal margin of the right ventral process of the spleen, was imaged in 23 platypuses (Figure 2B). Subsequent rotation of the transducer through approximately 90 degrees demonstrated this to be a cylindrical structure (Figure 2C), consistent with a blood vessel adjacent to the margin of the spleen. On two occasions, there was a smaller anechoic round structure seen adjacent to the above‐mentioned one, likely corresponding to an artery and vein in close proximity (Figure 2D).

**Figure 2:**
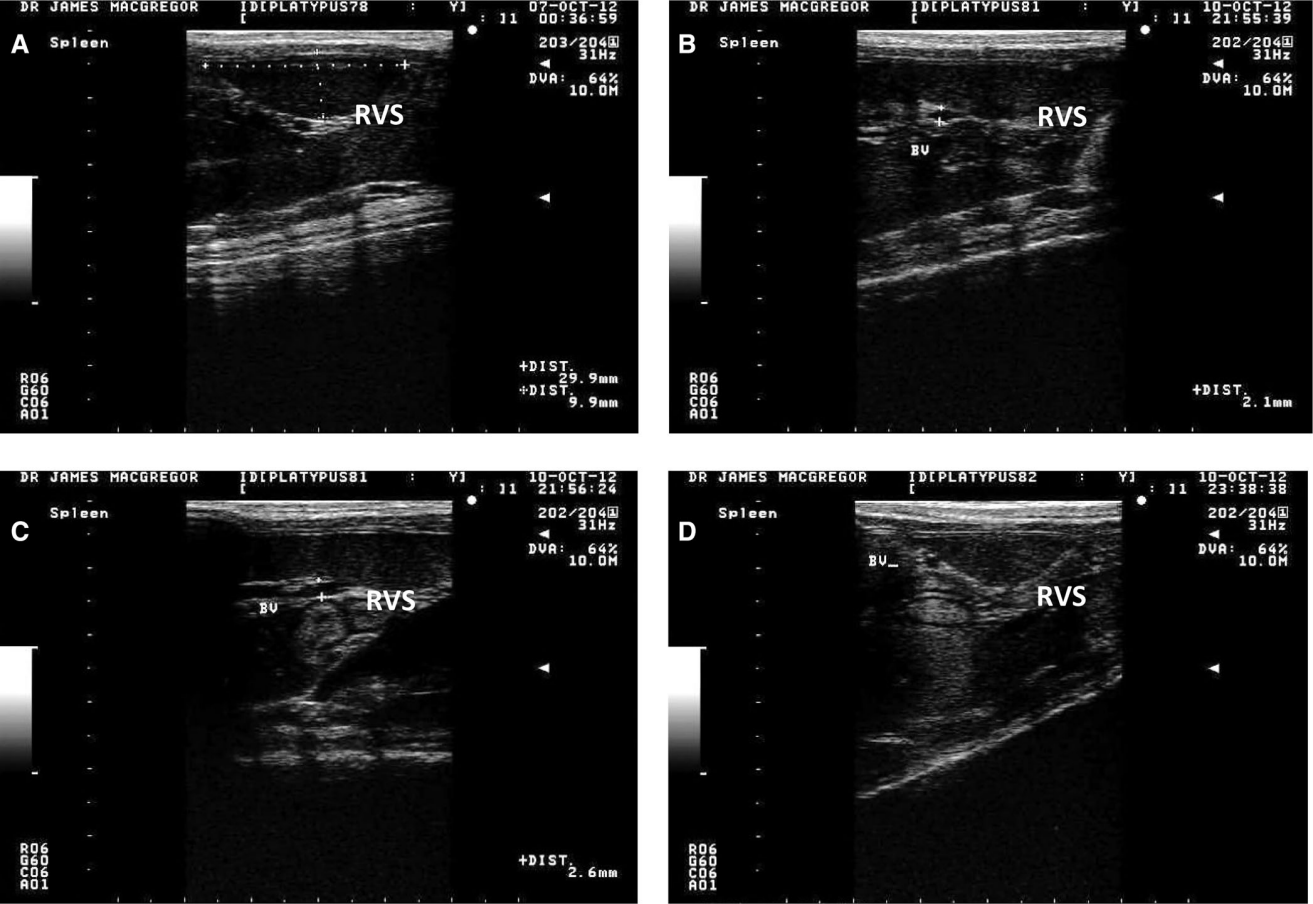
Ultrasound images of the platypus right ventral process of the spleen (RVS): (A) in sagittal oblique plane, (B) with associated blood vessel, (labelled “BV” with dorsal and ventral margin each indicated by +), in sagittal oblique plane, (C) with associated blood vessel, (labelled “BV” with dorsal and ventral margin each indicated by +), in transverse oblique plane, and (D) with two associated blood vessels, (labelled “BV”), in sagittal oblique plane. (cranial to left of sagittal oblique images).

The left kidney appeared as an ovoid‐shaped structure, with hyperechoic cortical and hypoechoic medullary regions in both its longitudinal and transverse axes (Figure 3). In this group of platypuses, it was always located dorsal to the right ventral process of the spleen (Figure 3A,B). The left kidney was at least partly positioned cranial to the spleen in 46 of these platypuses, directly dorsal to the spleen in two platypuses and at least partly caudal to the left kidney in six platypuses.

**Figure 3:**
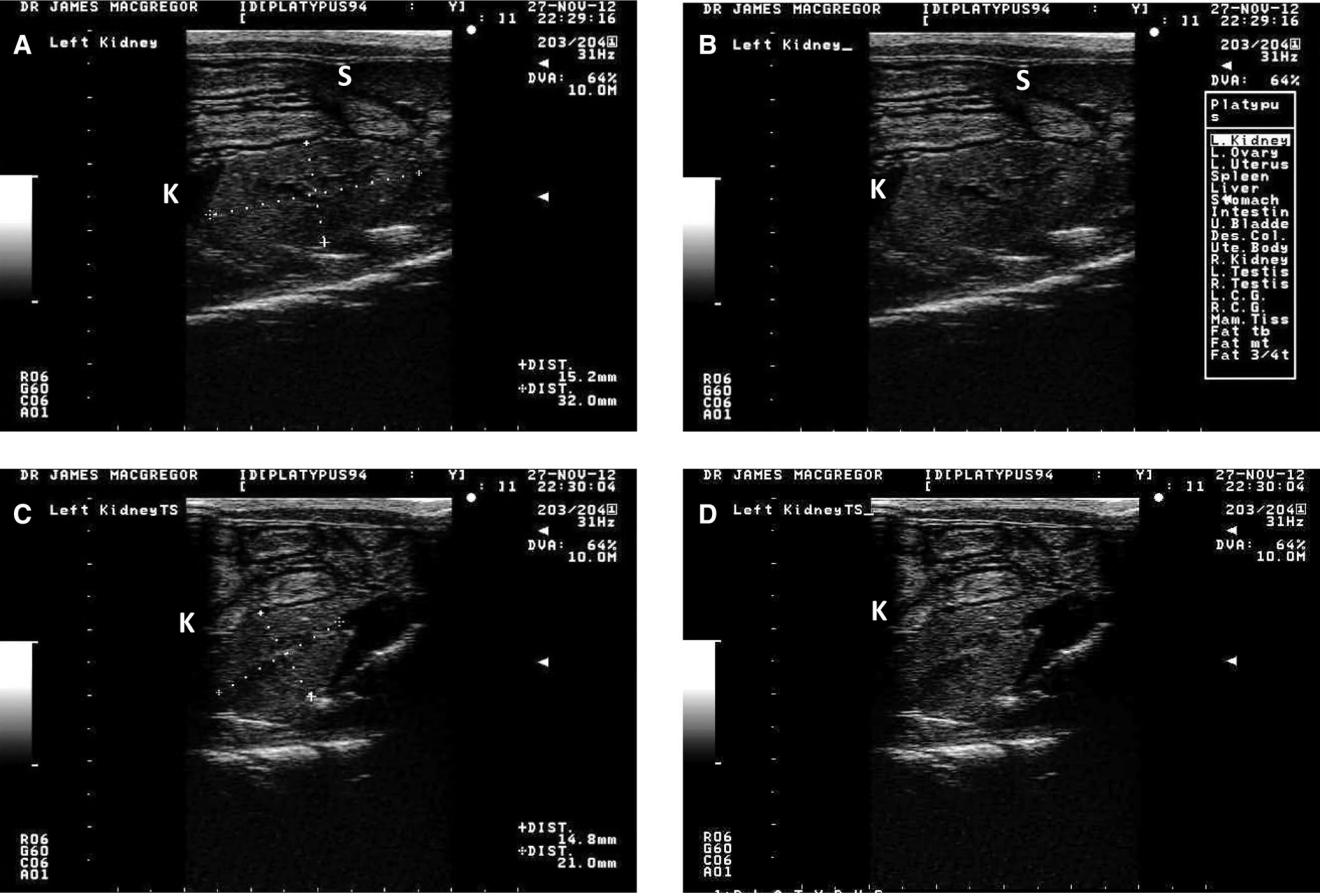
Ultrasound images of the platypus left kidney (K), (A) along its longitudinal axis (dotted lines indicate height and length) and showing position dorsal to the spleen (S), (B) the same image without dotted caliper lines, (C) left kidney along its transverse axis, (dotted lines indicate height and width) and (D) the same image without dotted caliper lines. In each image, intestinal structures are visible in the near field, ventral to the kidney.

In 17 platypuses, the left dorsal process of the spleen was imaged as a homogeneous, long structure, oriented craniocaudally, with a defined capsular margin, positioned adjacent to the dorsal body wall (Figure 4A). Due to the left ventral mid‐abdominal position of the clipped area, the urinary bladder was imaged as a mostly anechoic, fluid‐filled structure in the caudal abdomen, in only four individuals (two adult males, two adult females) (Figure 4B). Intestinal structures were frequently visible (Figure 3).

**Figure 4:**
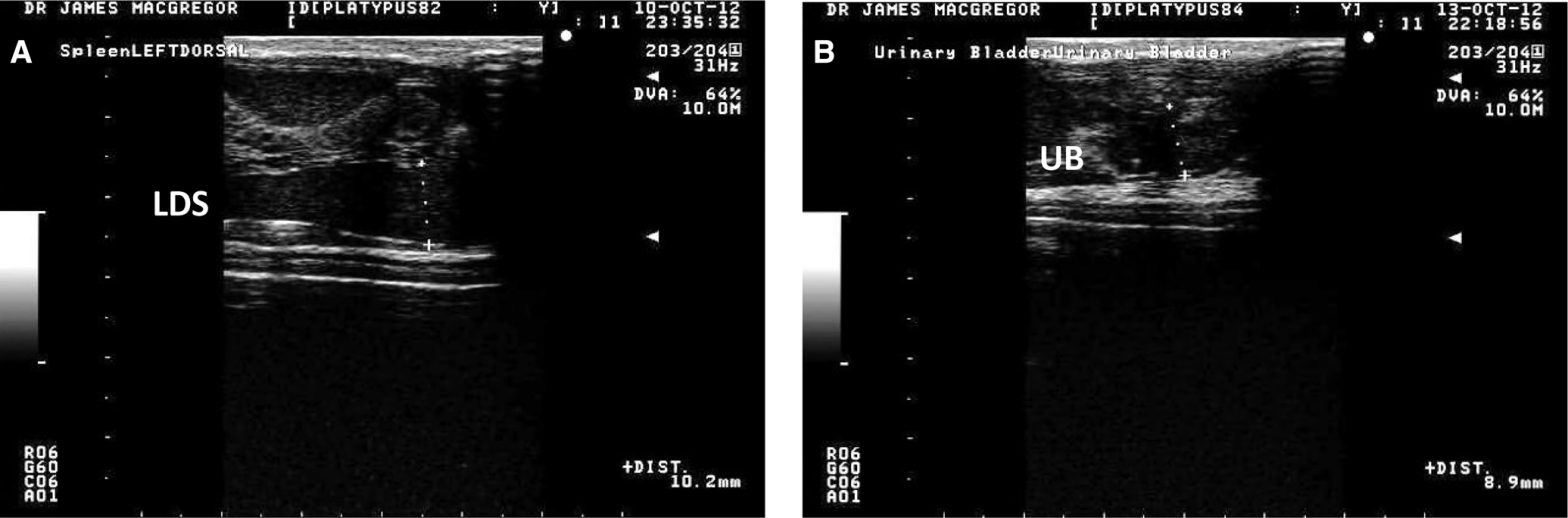
Ultrasound images of platypus (A) left dorsal process of spleen (LDS) in sagittal oblique plane, (dotted line indicates height), (B) urinary bladder (UB) in sagittal oblique plane, (dotted line indicates diameter).

The left testis was imaged as a homogeneous, encapsulated oval structure of intermediate echogenicity (isoechoic to the spleen), in both its longitudinal and perpendicular axes (Figure 5A–D). The left testis was located caudal or caudoventral to the left kidney and caudal or caudodorsal to the right ventral process of the spleen. It was possible to image the testis and kidney in the same 4 cm wide field of view in six platypuses (Figure 5G). The ovaries that were imaged in two females were located in a similar position to the testis in the male (Figure 5E,F). In each of these ovaries, one large developing follicle and one or more smaller follicles were imaged as hypoechoic structures. The surrounding ovarian tissue appeared as an oval structure (slightly more echogenic than the spleen), in both the sagittal oblique and transverse oblique planes.

**Figure 5:**
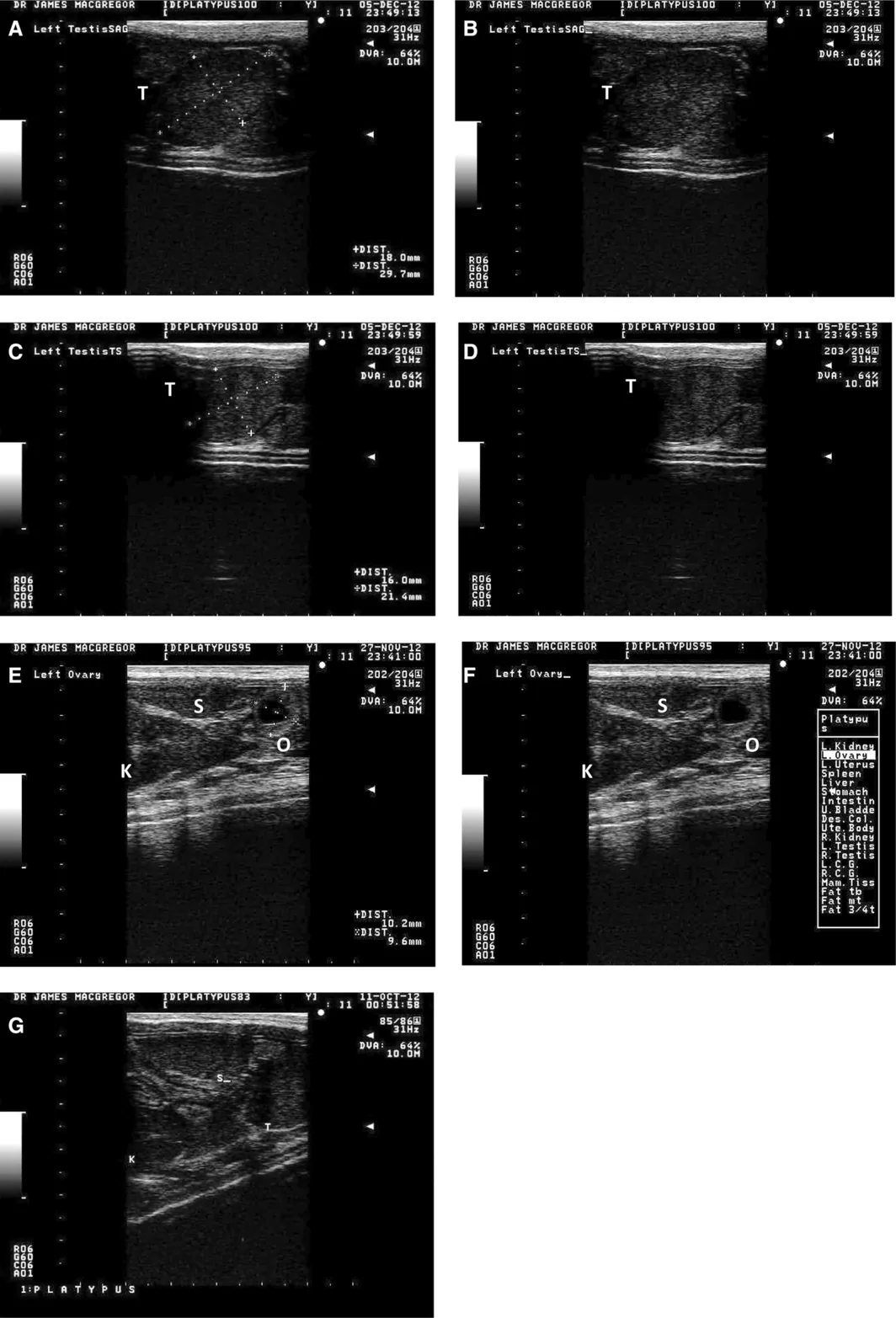
Ultrasound images of the platypus: (A) left testis (T; dotted lines showing length and thickness) along its longitudinal axis, (B) the same image without dotted calliper lines, (C) left testis (T; dotted lines showing width and thickness) along its transverse axis, (D) the same image without dotted calliper lines, (E) left ovary (O; dotted lines showing length and thickness) showing position in relation to spleen (S) and left kidney (K) and hypoechoic follicle, in sagittal oblique plane, (F) the same image without the dotted caliper lines and G) right ventral process of the spleen (S), left kidney (K) and left testis (T) in a single image in the sagittal oblique plane. (sagittal oblique images are oriented with the craniomedial end of the clipped ultrasound window at the left side of each image).

Of the measurements with results from greater than two individuals, the only distribution that showed a significant departure from normality was kidney length in females (*W* = 0.899, *p* = 0.028). Details of the dimensions of selected organs in the abdomen of adult platypuses from which appropriate images were captured are listed in Table 1. The distributions of left kidney lengths were significantly different for males and females, and the left kidney was significantly thicker in males than females. There was no statistical difference between the sexes for any of the splenic dimensions.

**Table 1:** The dimensions (cm) of abdominal organs and vessels. Sag = 30° to sagittal plane. Long = longitudinal axis of organ. Trans = transverse axis of organ (perpendicular to its longitudinal axis). * = more detail of seasonal variation provided in Macgregor.^18^ Dimensions whose distributions did not depart significantly from normality are described by mean, standard deviation, maximum and minimum. Dimensions whose distributions departed significantly from normality are expressed by median (underlined), interquartile range (underlined), maximum and minimum. Results of Mann–Whitney *U* tests between male and female distributions are listed as *U* and *p* values. Results of *t*‐tests between male and female distributions are listed as *t* value, degrees of freedom and *p* value. Statistically significant results in bold.

			Female					Male								
			Mean/median	Max	Min	SD/IQR	*n*	Mean	Max	Min	SD	*n*	*t* value	df	*U*	*p*
Left kidney	Length	Long	2.85	3.3	1.83	0.64	22	3.15	4.03	2.41	0.34	29			158.5	**0.002**
	Thickness	Long/trans	1.53	1.83	1.31	0.12	23	1.70	2.36	1.37	0.23	30	−3.21	51		**0.002**
	Width	Trans	2.45	2.48	2.41	0.05	2	2.43	2.89	2.05	0.32	11				
Left kidney – ventral process of spleen distance		Sag	0.38	1.04	0	0.25	20	0.61	1.09	0	0.26	26	−3.03	44		**0.004**
Left kidney – left testis dist.		Sag						1.13	1.97	0.1	0.83	6				
Left testis*	Length	Long						2.43	3.74	2.11	0.76	31				
	Thickness	Long/trans						1.46	2.2	0.55	0.45	31				
	Width	Trans						2.21	3.28	1.01	0.61	23				
Left kidney – left ovary dist.		Sag	1.18	1.18	1.18		1									
Left ovary*	Length	Sag	1.03	1.09	0.96	0.65	2									
	Thickness	Sag	1.06	1.09	1.02	0.35	2									
Ventral process of spleen	Width	Sag	2.42	3.27	1.14	0.38	21	2.56	3.26	1.87	0.37	26	−1.23	45		0.22
	Thickness	Sag	0.82	1.14	0.55	0.14	23	0.83	1.04	0.67	0.09	28	−0.32	49		0.75
Dorsal process of spleen	Thickness	Sag	0.88	1.12	0.59	0.18	10	0.97	1.26	0.72	0.20	6	−0.93	14		0.37
BV adjacent to vent. Spleen	Width	Sag	0.26	0.34	0.12	0.07	10	0.24	0.39	0.16	0.08	11	0.64	19		0.53
	Thickness	Sag	0.17	0.22	0.1	0.04	10	0.17	0.2	0.1	0.04	10	0.23	18		0.82

## Discussion

This study is the first detailed description of platypus abdominal ultrasonography. The collation of results from 54 individuals provides an important resource for veterinarians, particularly those who may only occasionally be required to examine a platypus, to assist in making evidence‐based decisions.

The exact approach to platypus abdominal ultrasonography, in particular in relation to eliminating air gaps between the transducer and the skin as well as the acoustic window used, is likely to depend on the reason for the examination. Platypus ultrasonography is made difficult by the thick, two layered fur which has evolved to hold air while underwater,^20^ as well as limited access between the bones of the skeleton.^7^, ^16^ This study used an acoustic window between the skeletal structures on the left side of the abdomen for reasons previously stated. We used a clipped area of skin before application of ultrasound gel to maximise image resolution, minimise examination time and to minimise heat loss during anaesthesia.^18^ Ongoing remote monitoring of these individuals has shown no evidence of an increase in attrition rate shortly after examination, and some of these individuals are still being monitored in the wild 13 years after their examinations^21^ (J Macgregor, unpublished data).

However, if additional and/or larger acoustic windows are required, further clipping is likely not to be appropriate. Alternatives to clipping include applying ultrasound gel or isopropyl alcohol or 70% ethanol, as reported by Barkman et al.^4^ in kangaroos, to the fur. Application of ultrasound gel to the fur without clipping would have a low effect on thermoregulation, however is likely to markedly reduce image resolution and working the gel into the thick fur would extend the duration of the examination by 10–20 min (Russell Jones, personal communication). Application of isopropyl alcohol or 70% ethanol to the fur would be quick and have no ongoing effect on thermoregulation, but would result in greater heat loss during the examination (particularly if used over a large area of skin). Furthermore, the presence of the thick, two layered fur is likely to reduce image resolution. All of these factors should be taken into account by the practitioner when deciding on the most appropriate approach for their situation.

The findings of this study were limited by two main factors. The first was the size of the acoustic window (reasons for which have been described above) which limited the imageable structures to those in the left side of the abdomen. The second factor was that the time available for ultrasonography in the broader field examinations was limited to a few minutes. As a result, detailed ultrasound investigations were limited to the left kidney, spleen and left testis/ovary. While the intestines were frequently present in the field of view (Figure 3), the urinary bladder was at times present (Figure 4(B)), and other structures such as the mesentery, peritoneum and lymph nodes would be expected to be visible, time precluded their investigation. A further limiting factor was that a doppler function, which would have assisted in investigation of vascular structures, was not available with the ultrasound unit used.

The left ovary was only identified when ovarian follicles were present. Opportunities to examine active ovaries are limited by the seasonal nature of their breeding^12^ and the fact that platypuses retire to a natal burrow for pregnancy and egg‐laying.^22^ Identification of the inactive ovary is complicated by it being of similar echogenicity to some surrounding structures, potentially being obscured by other structures,^23^ and in the platypus by it being only a few millimetres in width.^11^, ^14^ These factors, as well as the limited examination time described above lead to only two ovaries being identified.

This study has made a valuable contribution towards achieving a full characterisation of the sonographic appearance of the platypus abdomen – which currently remains incomplete in the literature. In addition to the measurement details listed in Table 1, we can summarise our observations to give the following guidelines for ultrasonography of the left side of the platypus abdomen:

- The described small acoustic window between the ribs and epipubic bone on the left ventral abdomen is appropriate for imaging a number of abdominal organs.
- The position of the right ventral process of the spleen in most platypuses, directly under the left ventral clipped area, makes this structure a good landmark and starting point for ultrasound examination of other organs from this acoustic window.
- With the right ventral process of the spleen as a starting point:
  ○ the left kidney can be found by scanning dorsally/cranially,
  ○ in males the left testis can be found by scanning caudally/dorsally (or caudally/ventrally from the left kidney),
  ○ in females, the left ovary may be found by scanning caudally/dorsally (in a similar position to the testis in males); however, it is likely to be small and challenging to image out of the breeding season during anoestrus,
  ○ the urinary bladder may be found by scanning caudally,
  ○ the left dorsal process of the spleen may be found dorsally.
- It should be noted that Tasmanian platypuses are, on average, bigger than those from other Australian states.^24^ As such, the dimensions of some organs of platypuses from other states may be smaller. In mainland states, to assist interpretation in a clinical setting, we would recommend comparing published external morphometric data of local platypuses to those in our study area.^24^

### Ethical statement

This study was approved by the Animal Ethics Committee of Murdoch University, Western Australia (Permit Number RW 2422/11), Department of Primary Industries, Parks, Water and Environment, Tasmania (Permit to take Wildlife for Scientific Purposes Numbers FA 11131 and FA 12165) and Inland Fisheries Service, Tasmania (Exemption Permit Number 2011‐10).

## Conflicts of interest and sources of funding

The authors declare no conflicts of interest. Funding was provided by Winifred Violet Scott Estate, a Holsworth Wildlife Research Endowment, a Caring For Our Country Community Action Grant [Project ID CAG 11‐00128], Central North Field Naturalists, the National Geographic Society [Grant C217‐12], the Cradle Coast Natural Resource Management, Tasmanian Alkaloids, the Australian Geographic Society, the Forestry Practices Authority, and the Edward Alexander Weston and Iris Evelyn Fernie Research Fund. We are grateful to the Forest Practices Authority, Tasmania, the University of Tasmania and the Tasmanian Department of Primary Industries, Parks, Water and Environment for the loan of fieldwork equipment. Open access publishing facilitated by Murdoch University, as part of the Wiley ‐ Murdoch University agreement via the Council of Australasian University Librarians.

## Data Availability

The data that support the findings of this study are available from the corresponding author upon reasonable request.
